# Gait changes with aging: an early warning sign for underlying pathology

**DOI:** 10.1007/s00415-025-12995-4

**Published:** 2025-03-07

**Authors:** Jorik Nonnekes, Erik Post, Gabriele Imbalzano, Bastiaan R. Bloem

**Affiliations:** 1 Radboud University Medical Centre; Donders Institute for Brain, Cognition and Behaviour; Department of Rehabilitation; Centre of Expertise for Parkinson & Movement Disorders, PO Box 9101, 6500 HB Nijmegen, The Netherlands; 2 Department of Rehabilitation, Sint Maartenskliniek, Nijmegen, The Netherlands; 3 Radboud University Medical Centre; Donders Institute for Brain, Cognition and Behaviour; Department of Neurology; Centre of Expertise for Parkinson & Movement Disorders, Nijmegen, The Netherlands; 4 Department of Neuroscience, Rita Levi Montalcini, ” University of Torino, Turin, Italy

**Keywords:** Gait, Senile gait, Aging, Mortality, Parkinson

## Abstract

Walking may appear to be a simple motor task, but is in fact a very complex behavior that involves virtually all levels of the nervous system. In daily clinical practice, subtle gait changes are commonly observed as we grow older, and these are often attributed to aging itself (the term “senile gait” was coined for this). However, growing evidence suggests that such age-related gait changes should not be regarded as a mere consequence of aging, but rather as indicators of underlying age-related disease. Numerous studies have shown that gait changes can be present for years during an otherwise prodromal phase of many progressive neurological disorders. As such, gait changes serve as clinical biomarkers of disease-related dysfunction in the neurological structures involved in gait control. We elaborate on the potential for gait to be exploited as an early warning system for underlying pathology. We also discuss the importance of such a proactive approach: an earlier diagnosis can lead to timely installment of symptomatic support, and sometimes start of prophylactic treatment. This can help reduce disability, and possibly increase survival because age-related gait disturbances are associated with increased mortality in the general population.

**Supplementary Information:**

The online version contains supplementary material available at 10.1007/s00415-025-12995-4.

## Introduction

Bipedal locomotion is one of the key features that distinguish humans from all other mammals. Even chimpanzees and gorillas (our closest primate relatives) do not walk on legs to move efficiently from one place to another. Walking may appear to be a simple, automatic activity that is conducted effortlessly; however, it is in fact a very complex behavior that involves almost all levels of the nervous system. Dysfunction in any of these levels can result in changes in gait. Initially such changes are often subtle: a slower walking speed, a reduced step length, or an increase in step width [1]. Those subtle changes are commonly interpreted as a mere consequence of normal aging (the term “senile gait” was coined for this). However, growing evidence suggests that such age-related gait changes should not be regarded as just a consequence of aging, but rather as indicators of underlying age-related disease. In this narrative review, we briefly summarize the requirements for normal gait control, elaborate on the scientific evidence pointing to gait changes as early markers of underlying disease, and highlight the potential for gait to be used as an early warning system for underlying pathology. Such an early detection can then serve as a basis for timely installment of symptomatic support, and sometimes start of prophylactic treatment.

## Gait control

Conceptually, gait involves three components: 1) rhythmic stepping movements, 2) balance control, and 3) the ability to adapt to environmental demands [2]. During stepping movements, balance – the control of the center of mass relative to the base of support, i.e., the feet in contact with the floor – is essential to prevent falling. This is challenging because the center of mass will fall outside the base of support with every step, making the position of subsequent foot placement crucial to restore the center of mass within the base of support [3]. The interplay between rhythmic stepping movements and control of the center mass needs to be adapted continuously to the environmental demands, both in a reactive manner (e.g., when stumbling) and a proactive manner (e.g., negotiating obstacles to prevent stumbling).

Physiologically, rhythmic stepping movements depend on a basic “locomotor network,” involving spinal central pattern generators, brainstem mesencephalic and cerebellar locomotor regions, and corticostriatal input projecting to the motor cortex [4]. For balance control, afferent sensory information (visual, vestibular, and proprioceptive) is crucial, which is integrated at the spinal, subcortical, and cortical level [5]. Distributed cortical areas, particularly the frontoparietal and supplementary motor areas, are involved in the adjustment and adaptation of walking [6]. Importantly, gait also depends on the peripheral nervous system (i.e., peripheral nerves and muscles), skeletal system (bones and joints), and cardiovascular fitness. Because gait is such a complex task, it is not surprising that dysfunction in any of the structures involved can result in changes in the gait pattern, which can be observed by careful clinical observation (box 1).

Box 1: assessment of gait in daily clinical practice Typically, walking needs to be examined in the corridor, as a patient should to be able to walk along a sufficiently long trajectory, ideally at least 10 m, and be able to make turns around the body axis. We always instruct patients to also walk with eyes closed, to take several steps walking backwards, and to run – all of these tests may offer valuable insights [7]. We recommend to observe the gait pattern both in the frontal and sagittal plane. It helps focus on spatiotemporal parameters and on specific gait phases. With respect to the spatiotemporal features, one needs to screen for abnormalities in gait speed, step height, step length, and regularity of step length and step width. Although spatiotemporal parameters cannot be quantified with the naked eye, deviations can be observed rather easily.

The gait cycle can be subdivided into gait phases. Using instrumented gait analysis, eight phases can be identified, but it is often difficult to identify these phases in real time, especially in patients with neurological gait impairment. A more practical approach is to subdivide the gait cycle into the stance phase, the swing phase of each leg, and the transitions between those phases. It helps identify these phases for the right and left leg separately and screen whether any abnormalities exist. We recommend to also focus on the trunk and arms, as these provide valuable information as well. It should be noted that arm movements may differ in specific ways between regular walking and running [8].

Finally, it helps not only focus on visual information but also listen to the sound of footfall [7]; for example, a reduced foot clearance creates a shuffling sound, foot elevator weakness produces a flapping sound, and asymmetry between subsequent steps results in an irregular footfall.

## Gait as an early marker of disease

When the concept of senile gait was first introduced, it was generally felt that the process of aging itself was a sufficient explanation for the changes in walking [9]. Such a senile gait was thought to consist of some general slowing in walking speed, widening of the base of support, and an ataxic staggering [10]. The implicit message behind this construct was that there was no need for any ancillary testing in older individuals who presented with merely such changes in gait, nor was there any reason to initiate symptomatic treatment. However, this concept has progressively been abandoned over the last two decades. Indeed, numerous studies have now shown that changes in gait can be the earliest clinical sign of what later becomes a full-blown progressive neurological disorder [11–14]. Moreover, it has become clear that changes in gait with aging are all but benign, as they are associated with an increased mortality in the general population, with death being related either directly to the underlying neurodegenerative condition, or indirectly because of a secondary lack of mobility [15, 16].

Finally, gait can remain fully conserved in even very old persons, suggesting that changes in gait are not an inevitable companion of aging in otherwise very healthy persons [10].

### Cerebrovascular pathology

**Fig. 1:**
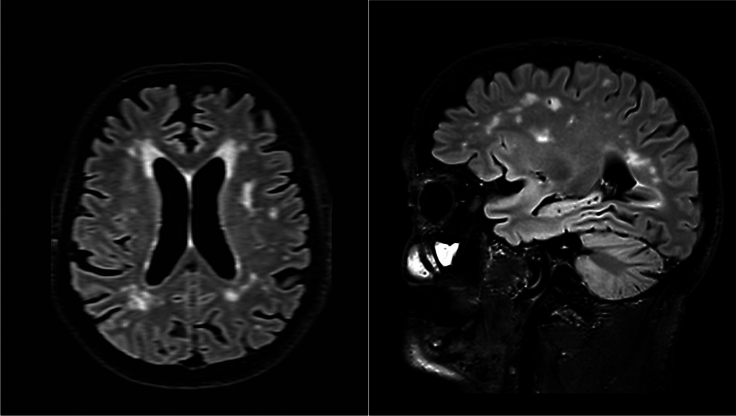
Axial and sagittal views from T2-weighted FLAIR sequences of a 1.5 Tesla MRI scan obtained from the 81-year old man. The images reveal diffuse hyperintensities predominantly located in the periventricular and subcortical regions, consistent with ischemic white matter changes characteristic of chronic small vessel disease, commonly seen in older adults with cardiovascular risk factors

This presumably constitutes the most common type of underlying neurological disease in elderly persons presenting with isolated changes in their gait. Longitudinal follow-up studies have shown an increased risk of dying from cardiovascular complications in such individuals, which is an indirect suggestion an underlying cardiovascular disease [15]. Neuroimaging studies using either CT or MRI scans have shown that persons with age-related changes in gait commonly have white matter lesions or lacunar infarctions [17], see video 1 and Fig. 1 for an illustrative case.

### Parkinson

Another underlying neurological disorder to consider is Parkinson’s disease (PD). It is now clear that gait changes can be present during what is otherwise a prodromal phase of PD, the diagnosis of which is made only years later after additional signs have emerged. The first line of evidence stems from two studies involving first-degree relatives of individuals with PD with a LRRK2-G2019S mutation [13, 14]. In the first study, 52 healthy relatives were included, of whom 27 were non-carriers and 25 were carriers of the LRRK2-G2019S mutation. Importantly, the carriers did not yet fulfill the diagnostic criteria for PD [14]. Results indicated that stride time variability was higher among carriers compared to non-carriers, both under dual-task conditions and when walking at a fast pace. Moreover, the amplitude of the dominant peak of the accelerometer signal was reduced in carriers compared to non-carriers. In a subsequent larger study involving 186 relatives of individuals with PD with a LRRK2-G2019S mutation (122 of whom were carriers), greater arm swing asymmetry and variability during dual-task conditions were observed in the carriers, as well as lower axial rotation smoothness [13].

A second line of evidence involves individuals with another prodromal sign of PD, namely, those with an idiopathic REM sleep behavior disorder (IRBD). In the first study, 78 persons with IRBD were included, of whom 20 developed parkinsonism during the course of the study [18]. Gait changes (measured using items 28–31 of the MDS-UPDRS) occurred on average 4.4 years prior to the diagnosis of parkinsonism. A larger study involving 154 patients with IRBD (of whom 55 converted to parkinsonism or dementia) showed that subjective gait changes (measured using the MDS-UPDRS part II) were present six years prior to phenoconversion, and objective gait changes (measured using the MDS-UPDRS part III) two years before [19].

A third line of evidence stems from prospective cohort studies within the general population. One study included 696 healthy adults (mean age 63 years at the time of inclusion), who underwent assessments four times at two-year intervals [20]. During these assessments, participants walked at different speeds under single- and dual-task conditions, with a wearable sensor attached to their lumbar region. Sixteen participants were diagnosed with PD during the course of the study, on average 4.5 years after the first visit. Results showed that higher step time variability and asymmetry in all gait characteristics were associated with a shorter time to diagnosis. These findings are in line with a nested case–control study from the prospective Rotterdam Study, which involved 109 individuals who developed PD and 1.199 age- and sex-matched controls [21]. Gait changes (scored by an assessor on a 3-point scale) occurred in the last 4.5 years prior to diagnosis, but importantly, a reduced arm swing (also scored by an assessor on a 3-point scale) was measured 8.6 years prior to diagnosis. The latter finding was in line with earlier anecdotal work that had already suggested that an asymmetrically reduced arm swing could be the very first sign of what later becomes full-blown PD [11, 12].

A recent cohort study used a subset of the UK Biobank study and focused on data collected using a wrist-worn accelerometer [22]. The accelerometer data are influenced by arm swing during gait, but obviously also by other activities. A strength of this study was its scale: 103.712 participants wore the accelerometer, of whom 196 individuals were diagnosed with PD more than two years after the accelerometry data collection. Interestingly, in the prodromal phase, these individuals had significantly reduced acceleration profiles compared with their age- and sex-matched controls. Moreover, no other modality (e.g., genetics, lifestyle determinants, blood samples, or prodromal symptoms) was a better predictor of the future diagnosis of PD, up to seven years prior to diagnosis.

Taken together, these various studies suggest that gait changes may occur two to seven years prior to the diagnosis of PD. These gait changes become more prominent during challenging situations (e.g., when performing a concurrent cognitive task), and involve asymmetry, reduced acceleration, and increased variability. This recognition can have implications. Of course, at the moment, a diagnosis of PD cannot be made based on an isolated change in gait alone, nor will it lead to immediate changes in treatment, other than the advice to remain physically active. However, timely recognition of such early changes in gait, alongside detection of other prodromal signs (whether these be clinical signs such as IRBD, constipation or hyposmia, or a diagnostic biomarker such as an alpha synuclein assay), may lead to a more reliable detection of the prodromal phase of PD. Such individuals can be offered the opportunity to participate in one of the many ongoing trials that are investigating the efficacy of disease-modifying interventions that aim to slow down the process of neurodegeneration, and thereby delay or perhaps even postpone the moment of diagnosing full-blown PD.

### Inherited movement disorders

Prodromal gait changes are also seen in other progressive movement disorders. Examples include changes in gait that have been identified in otherwise presymptomatic individuals with a genetically determined predisposition to Huntington disease, spinocerebellar ataxia (SCA), hereditary spastic paraplegia (HSP), or X-linked dystonia-parkinsonism. We here summarize the main findings. One study assessed gait in 15 non-manifesting mutation carriers of the Huntington gene compared to 20 healthy controls. Participants were instructed to walk at their preferred speed on a computerized walkway, which recorded spatiotemporal variables. The results showed that compared to controls, those genetically predisposed to Huntington disease manifested a decreased gait speed and stride length, increased double support time, and higher gait variability [23]. Another study included 30 individuals with pathogenic variants in the SPAST gene (associated with HSP type 4 (SPG4)), and compared their gait to 23 healthy controls. Gait was assessed in a movement laboratory using an infrared-camera-based motion capture system, and participants were instructed to walk multiple times at their preferred speed across a 10-m walkway [24]. No significant differences in spatiotemporal parameters were observed between both groups. However, kinematic analysis using an infrared-camera-based motion capture system revealed a reduced maximum heel ground clearance and range of motion of the foot segment during the gait cycle in the prodromal carriers of the SPAST gene. A study that quantified gait in 30 asymptomatic carriers of an expanded ATXN2 allele (≥ 32 CAG repeats; associated with SCA2) and 30 matched controls found several differences between both groups, including larger variability of the swing period in the presymptomatic carriers [25]. Finally, one study showed that gait changes (expressed by a data-driven combination of 32 features measured via 6 wearable sensors) precede the onset of fully manifest X-linked dystonia-parkinsonism [26].

### Cognitive impairment

There is abundant evidence that a reduced gait speed precedes and predicts cognitive impairment [27–31]. A recent multicohort study involving 1.570 eligible participants confirmed this pattern [32]. All participants had no dementia at enrollment and were aged 65 years or older; they consented to annual clinical assessments and post-mortem brain donation. Gait function was assessed by asking participants to walk 8 feet and perform two 360-degree turns. The time to complete the trajectory and the required number of steps were used to construct a composite gait score. Gait decline preceded cognitive decline by up to a decade in these participants. Interestingly, autopsy revealed that macroinfarcts, rather than Alzheimer’s disease pathology, were associated with declining gait function that preceded cognitive decline. This would be in line with the association between age-related gait changes and underlying cerebrovascular pathology that we mentioned earlier.

### Polyneuropathy

Gait changes are often the first presenting sign in patients with polyneuropathy, and the prevalence of polyneuropathy increases with age [33]. Due to sensory impairments and subsequent reduced balance capacity, people with polyneuropathy typically walk with a broadened base of support, reduced gait speed and increased double support phase. In the above-mentioned population-based Rotterdam Study, 1.445 participants were screened for polyneuropathy (using a symptom questionnaire, neurological examination and nerve conduction studies) [34]. People with definite polyneuropathy were almost two times more likely to have fallen in the preceding year, and polyneuropathy was related to falls that resulted in injury. In a random subset of 977 participants, gait was assessed with an electronic walkway; people with polyneuropathy exhibited reduced gait speed, reduced cadence, and more errors tandem walking.

### Osteoarthritis

Although not a neurological cause of gait impairment, it is important to mention the influence of musculoskeletal conditions such as osteoarthritis on gait. Gait speed is reduced in patients with osteoarthritis of the hip[35, 36] or knee [37]. In both groups, this difference stems from a shortened stride length combined with a reduced cadence [35, 38]. Importantly, osteoarthritis usually results in an asymmetrical gait pattern, with a smaller step length and shorted stance duration on the (most) affected site (i.e., antalgic gait).

### Prognostic implications

One important reason why age-related changes in gait merit our attention is that these are associated with an increased mortality risk. Specifically, several prospective cohort studies focused on the relationship between gait and survival. The relationship between gait changes (e.g., a reduced walking speed) and an increased mortality risk has been demonstrated convincingly by several studies. For example, in a population-based longitudinal study, 126 elderly individuals (87–97 years old) were classified as having either a normal gait, a senile gait (i.e., changes in walking that could not readily be explained by overt pathology), or a gait disorder caused by known disease [15]. Mortality risk (mainly from cardiovascular disease) was higher in those with a senile gait compared to those with a normal gait, but similar to those with a gait disorder caused by known disease. Another study pooled the data from nine cohort studies involving 34.485 adults aged 65 year or older, who were followed for 6 to 21 years [16]. Across all nine studies, a slower gait speed was associated with a reduced survival. Specifically, for every 0.1 m/s increase in walking speed, there was a corresponding decrease in mortality risk (hazard ratio per 0.1 m/s, 0.88). Others made similar observations. In the Health, Aging, and Body Composition Study, gait speed was periodically measured in 2.364 initially healthy elderly individuals over an 8-year period during a 20-m walk [39]. Changes in gait speed were classified into three categories: slow, moderate, and fast decline. Those with a fast decline in gait speed had a 90% greater risk of mortality compared to those with a slow decline. In the aforementioned Rotterdam Study, 4.490 participants underwent a gait assessment and were followed up [40]. The mean follow-up duration between the gait assessment and the mortality or censor date was 4.5 years. Gait was assessed with an electronic walkway of approximately 6 m long. An electronic walkway has the advantage of collecting not only gait speed but also other spatiotemporal parameters. These parameters were classified into seven domains. A worse pace (primarily reflecting gait speed) was most strongly associated with mortality risk: a 0.1 m/s decrease in gait speed was associated with a 1.21 times higher hazard of mortality, followed by worse rhythm (gait variables involving temporal gait measures), worse phases (gait variables on the ratio between single and double support times), and a widened base of support.

## Implications for daily clinical practice and future studies

One important practical implication is that in daily clinical practice, gait changes should not be automatically accepted as a normal consequence of aging, but rather as a “clinical biomarker” of underlying disease-related dysfunction of one or more of the (neurological) structures involved in gait control. Not rarely, the gait disorder is the result of multiple and each relatively minor impairments, of which the sum is greater than each of its parts (i.e., a patient might both have a mild polyneuropathy, and white matter lesions, both of which would have remained largely inconspicuous, but which together can lead to noticeable gait problems). We should point out that the age-related gait changes may be sensitive to the presence of underlying pathology, but they are typically not specific. A good example is the reduced gait speed, which is not specific for any single neurological condition, but is rather observed more generically in the prodromal phase of many different neurological conditions. This is understandable, because reducing one’s gait speed is a readily applicable adaptation to decrease the complexity of balance control during walking. The same applies to widening of the base of support, which is again a commonly seen adaptation in response to a self-perceived deficit in gait or balance [1]. However, other signs are not the result of compensatory adjustments but do reflect the primary deficits caused by the underlying pathology. An example is unilaterally reduced arm swing, which has a limited differential diagnosis, including an early stage of parkinsonism. We have previously introduced a sign-based approach to neurological gait changes [1]. According to this sign-based approach, we describe that once gait abnormalities have been identified, one can use their presenting signs as a starting point to produce a focused differential diagnosis, which starts with a detailed clinical workup. Specifically, one can use history taking and targeted clinical tests to narrow down the differential diagnosis. An example of such a clinical test is the Romberg test, which can help differentiate between cerebellar and sensory ataxia [1]. Using this approach, one is often able to find the underlying substrate, but sometimes waiting for progression over time (monitored using follow-up consultations) is needed to establish a diagnosis. Patient education can also play a role here, by instructing affected individuals to monitor for progression of gait disability, or the emergence of other alarm signals, such as urinary urgency or cognitive decline, which could lead to an earlier re-examination.

Subtle gait changes may thus help clinicians in reaching an earlier diagnosis. This is in general a service for most affected individuals, who already perceived the changes in their gait much earlier (sometimes even years before), and who are now offered an explanation. And importantly, a timely diagnosis also opens the opportunity for early intervention. One example is optimal cardiovascular management in individuals with gait changes due to white matter lesions. At the least, such individuals should be encouraged to remain physically active despite their age-related gait disorders – it could well be that the poor prognosis is explained at least in part by a resultant secondary immobility. For many other neurological disorders, an early intervention is not yet possible, but there are many ongoing studies worldwide that are evaluating promising therapies that have disease-modifying potential [41]. As we mentioned earlier, early occurring gait changes might well become part of a larger test battery that can identify individuals in the prodromal phase of their disease, offering them the opportunity to participate in research studies that explore the possibilities for disease modification in an early disease phase when there is still much neuronal tissue to be rescued. The ability to detect even subtle gait changes using technological advances is promising in this regard. This includes a detailed gait analysis in state-of-the-art gait laboratories, where all elements of gait can be quantified in great detail. Moreover, some of the studies highlighted above suggest that such a sophisticated gait analysis is not always necessary to identify changes; several gait features can already be detected using a wrist-worn accelerometer (e.g., integrated within a smartwatch) [22]. These developments create exciting new opportunities to utilize gait as an early warning system at the population level.

## Search strategy

We searched PubMed for relevant articles published in English from database inception to July, 2024. Potential papers were identified with the terms “gait disorder,” “senile gait,” “wearables,” “survival rate,” “gait in elderly,” “ageing,’’ or any of the following specific movement disorders: “Parkinson’s disease,” “Parkinsonism,” “Lewy body dementia,” “Ataxia,” “Huntington’s disease,” “HSP,” “Alzheimer’s disease,” “Vascular dementia,” “Cognitive impairments,” “Small vessel disease,” “Normal Pressure Hydrocephalus,” “Osteoarthritis,” “Polyneuropathy,” “Charcot-Mary-Tooth,” “Chorea,” “Dystonia,” and “sensory impairments.” Selected articles were also identified by examining reference lists of found articles and the publication list of the first and final author of each publication.

## Supplementary Information

Below is the link to the electronic supplementary material.

Supplementary file1 (MOV 3867 KB)

## References

[1] Nonnekes J et al (2018) Neurological disorders of gait, balance and posture: a sign-based approach. Nat Rev Neurol 14(3):183–189 29377011 10.1038/nrneurol.2017.178

[2] Balasubramanian CK, Clark DJ, Fox EJ (2014) Walking Adaptability after a Stroke and Its Assessment in Clinical Settings. Stroke Res Treatment 2014:1–21 10.1155/2014/591013 PMC4164852 25254140

[3] Bruijn SM, van Dieën JH (2018) Control of human gait stability through foot placement. J Royal Soc Interface 15(143):20170816 10.1098/rsif.2017.0816 PMC6030625 29875279

[4] Nutt JG et al (2011) Freezing of gait: moving forward on a mysterious clinical phenomenon. Lancet Neurol 10(8):734–744 21777828 10.1016/S1474-4422(11)70143-0 PMC7293393

[5] Forbes PA, Chen A, Blouin JS (2018) Sensorimotor control of standing balance. Handb Clin Neurol 159:61–83 30482333 10.1016/B978-0-444-63916-5.00004-5

[6] Takakusaki K (2023) Gait control by the frontal lobe. Handb Clin Neurol 195:103–126 37562865 10.1016/B978-0-323-98818-6.00021-2

[7] Snijders AH et al (2007) Neurological gait disorders in elderly people: clinical approach and classification. Lancet Neurol 6(1):63–74 17166803 10.1016/S1474-4422(06)70678-0

[8] Fearon C et al (2024) Arm swing while walking and running: a new clinical feature to separate parkinson’s disease from functional parkinsonism. Mov Disord Clin Pract 11(2):166–170 38169144 10.1002/mdc3.13952 PMC10883393

[9] Critchley M (1948) On senile disorders of gait, including the so-called senile paraplegia. Geriatrics 3(6):364–370 18893756

[10] Bloem BR et al (1992) Investigation of gait in elderly subjects over 88 years of age. J Geriatr Psychiatr Neurol 5(2):78–84 10.1177/002383099200500204 1590914

[11] Lees AJ (1992) When Did Ray Kennedy Parkinsons-Disease Begin. Mov Disord 7(2):110–116 1584235 10.1002/mds.870070203

[12] Nurnberger L et al (2015) Ultrasound-based motion analysis demonstrates bilateral arm hypokinesia during gait in heterozygous PINK1 mutation carriers. Mov Disord 30(3):386–392 25545816 10.1002/mds.26127

[13] Mirelman A et al (2016) Arm swing as a potential new prodromal marker of Parkinson’s disease. Mov Disord 31(10):1527–1534 27430880 10.1002/mds.26720 PMC5053872

[14] Mirelman A et al (2011) Gait Alterations in Healthy Carriers of the LRRK2 G2019S Mutation. Ann Neurol 69(1):193–197 21280089 10.1002/ana.22165

[15] Bloem BR et al (2000) Idiopathic senile gait disorders are signs of subclinical disease. J Am Geriatr Soc 48(9):1098–1101 10983910 10.1111/j.1532-5415.2000.tb04786.x

[16] Studenski S et al (2011) Gait speed and survival in older adults. JAMA 305(1):50–58 21205966 10.1001/jama.2010.1923 PMC3080184

[17] Inzitari D et al (2009) Changes in white matter as determinant of global functional decline in older independent outpatients: three year follow-up of LADIS (leukoaraiosis and disability) study cohort. Bmj-British Med J 339:b2477 10.1136/bmj.b2477 PMC2714680 19581317

[18] Postuma RB et al (2012) How does parkinsonism start? Prodromal parkinsonism motor changes in idiopathic REM sleep behaviour disorder. Brain 135:1860–1870 22561644 10.1093/brain/aws093

[19] Fereshtehnejad SM et al (2019) Evolution of prodromal Parkinson’s disease and dementia with Lewy bodies: a prospective study. Brain 142(7):2051–2067 31111143 10.1093/brain/awz111

[20] Del Din S et al (2019) Gait analysis with wearables predicts conversion to parkinson disease. Ann Neurol 86(3):357–367 31294853 10.1002/ana.25548 PMC6899833

[21] Darweesh SK et al (2017) Trajectories of prediagnostic functioning in Parkinson’s disease. Brain 140(2):429–441 28082300 10.1093/brain/aww291

[22] Schalkamp AK et al (2023) Wearable movement-tracking data identify Parkinson’s disease years before clinical diagnosis. Nat Med 29(8):2048–2056 37400639 10.1038/s41591-023-02440-2

[23] Rao AK et al (2008) Spectrum of gait impairments in presymptomatic and symptomatic Huntington’s disease. Mov Disord 23(8):1100–1107 18412252 10.1002/mds.21987

[24] Lassmann C et al (2022) Specific Gait Changes in Prodromal Hereditary Spastic Paraplegia Type 4: preSPG4 Study. Mov Disord 37(12):2417–2426 36054444 10.1002/mds.29199

[25] Velazquez-Perez L et al (2021) Prodromal spinocerebellar ataxia type 2 subjects have quantifiable gait and postural sway deficits. Mov Disord 36(2):471–480 33107647 10.1002/mds.28343

[26] Steinhardt J et al (2022) Prodromal X-Linked Dystonia-Parkinsonism is Characterized by a Subclinical Motor Phenotype. Mov Disord 37(7):1474–1482 35491955 10.1002/mds.29033

[27] Verghese J (2021) Motoric cognitive risk syndrome: Next steps. Eur J Neurol 28(8):2467–2468 34057808 10.1111/ene.14949

[28] Camicioli R et al (1998) Motor slowing precedes cognitive impairment in the oldest old. Neurology 50(5):1496–1498 9596020 10.1212/wnl.50.5.1496

[29] Buracchio T et al (2010) The trajectory of gait speed preceding mild cognitive impairment. Arch Neurol 67(8):980–986 20697049 10.1001/archneurol.2010.159 PMC2921227

[30] Stephan Y et al (2024) Balance, Strength, and Risk of Dementia: Findings From the Health and Retirement Study and the English Longitudinal Study of Ageing. J Gerontol A Biol Sci Med Sci 79(8):glae165 38918945 10.1093/gerona/glae165 PMC11249972

[31] Kueper JK et al (2017) Motor function and incident dementia: a systematic review and meta-analysis. Age Ageing 46(5):729–738 28541374 10.1093/ageing/afx084

[32] Oveisgharan S et al (2024) The time course of motor and cognitive decline in older adults and their associations with brain pathologies: a multicohort study. Lancet Healthy Longevity 5(5):e336–e345 38582095 10.1016/S2666-7568(24)00033-3 PMC11129202

[33] Hanewinckel R et al (2016) Prevalence of polyneuropathy in the general middle-aged and elderly population. Neurology 87(18):1892–1898 27683845 10.1212/WNL.0000000000003293

[34] Hanewinckel R et al (2017) Polyneuropathy relates to impairment in daily activities, worse gait, and fall-related injuries. Neurology 89(1):76–83 28566544 10.1212/WNL.0000000000004067

[35] Constantinou M et al (2014) Spatial-temporal gait characteristics in individuals with hip osteoarthritis: a systematic literature review and meta-analysis. J Orthop Sports Phys Ther 44(4):291-B7 24450373 10.2519/jospt.2014.4634

[36] Constantinou M et al (2017) Hip joint mechanics during walking in individuals with mild-to-moderate hip osteoarthritis. Gait Posture 53:162–167 28167387 10.1016/j.gaitpost.2017.01.017

[37] Mills K, Hunt MA, Ferber R (2013) Biomechanical deviations during level walking associated with knee osteoarthritis: a systematic review and meta-analysis. Arthritis Care Res (Hoboken) 65(10):1643–65 23554153 10.1002/acr.22015

[38] Boekesteijn RJ et al (2021) Independent and sensitive gait parameters for objective evaluation in knee and hip osteoarthritis using wearable sensors. BMC Musculoskelet Disord 22(1):242 33658006 10.1186/s12891-021-04074-2 PMC7931541

[39] White DK et al (2013) Trajectories of gait speed predict mortality in well-functioning older adults: the Health, Aging and Body Composition study. J Gerontol A Biol Sci Med Sci 68(4):456–64 23051974 10.1093/gerona/gls197 PMC3593620

[40] Dommershuijsen LJ et al (2020) Unraveling the association between gait and mortality-one step at a time. J Gerontol Series a-Biol Sci Med Sci 75(6):1184–1190 10.1093/gerona/glz282 PMC7243583 31807749

[41] Oosterhof TH et al (2024) Considerations on How to Prevent Parkinson’s Disease Through Exercise. J Parkinsons Dis 14(s2):S395–S406 39031383 10.3233/JPD-240091 PMC11492051

